# Deciphering the tumor microenvironment through radiomics in non-small cell lung cancer: Correlation with immune profiles

**DOI:** 10.1371/journal.pone.0231227

**Published:** 2020-04-06

**Authors:** Hyun Jung Yoon, Jun Kang, Hyunjin Park, Insuk Sohn, Seung-Hak Lee, Ho Yun Lee

**Affiliations:** 1 Department of Radiology and Center for Imaging Science, Samsung Medical Center, Sungkyunkwan University School of Medicine, Seoul, South Korea; 2 Department of Radiology, Veterans Health Service Medical Center, Seoul, South Korea; 3 Department of Hospital Pathology, Seoul St. Mary’s Hospital, College of Medicine, The Catholic University of Korea, Seoul, South Korea; 4 School of Electronic and Electrical Engineering, Sungkyunkwan University, Suwon, South Korea; 5 Center for Neuroscience Imaging Research, Institute for Basic Science, Suwon, South Korea; 6 Statistics and Data Center, Samsung Medical Center, Seoul, South Korea; 7 Department of Electrical and Computer Engineering, Sungkyunkwan University, Suwon, South Korea; 8 Department of Health Sciences and Technology, SAIHST, Sungkyunkwan University, Seoul, South Korea; Peter MacCallum Cancer Centre, AUSTRALIA

## Abstract

Growing evidence suggests that the efficacy of immunotherapy in non-small cell lung cancers (NSCLCs) is associated with the immune microenvironment within the tumor. We aimed to explore radiologic phenotyping using a radiomics approach to assess the immune microenvironment in NSCLC. Two independent NSCLC cohorts (training and test sets) were included. Single-sample gene set enrichment analysis was used to determine the tumor microenvironment, where type 1 helper T (Th1) cells, type 2 helper T (Th2) cells, and cytotoxic T cells were the targets for prediction with computed tomographic (CT) radiomic features. Multiple algorithms were in the modeling followed by final model selection. The training dataset comprised 89 NSCLCs and the test set included 60 cases of lung squamous cell carcinoma and adenocarcinoma. A total of 239 CT radiomic features were used. A linear discriminant analysis model was selected for the final model of Th2 cell group prediction. The area under the curve value of the final model on the test set was 0.684. Predictors of the linear discriminant analysis model were skewness (total and outer pixels), kurtosis, variance (subsampled from delta [subtraction inner pixels from outer pixels]), and informational measure of correlation. The performances of radiomics on test set of Th1 and cytotoxic T cell were not accurate enough to be predictable. A radiomics approach can be used to interrogate an entire tumor in a noninvasive manner and provide added diagnostic value to identify the immune microenvironment of NSCLC, in particular, Th2 cell signatures.

## Introduction

Immune checkpoint blockade therapy, an anti-cancer treatment that potentiates the ability of the immune system to recognize and destroy cancer cells, has become a standard in the care of patients with non-small cell lung cancers (NSCLCs) [1–5]. The expression of the programmed death-ligand 1 (*PD-L1*) gene in the tumor has been associated with a response to checkpoint blockade therapy and prognosis, to some extent [6, 7], but the understanding of the complex interactions between tumors and their microenvironment remains insufficient to distinguish between patients who will respond to therapy and those who should be offered alternative treatment. Growing evidence suggests that the efficacy of immunotherapy in cancers is associated with the immune microenvironment of tumors, thereby type 1 and 2 immune responses are believed to be important predictive biomarkers [8–11]. Generally, type 1 immune responses are thought to be the most relevant to antitumor immunity, and many cancer immunotherapies are designed to augment type 1 immune responses that involve cytotoxic T cells and type 1 helper T (Th1) cells to eliminate tumors [9], whereas a type 2 immune response characterized by type 2 helper T (Th2) cells is often associated with a tumor-permissive environment [8]. Recent work revealed that the inhibition of melanoma tumor formation occurred in an activated type 2 immune response that was distinct from the classical tumor microenvironment [10].

Immunohistochemistry after tumor resection is the only technique that yields quantitative information on the immune microenvironment and immune response type assessment. Therefore, efficient and reliable noninvasive biomarkers are urgently needed. Radiomics has recently been identified as a way to use accurate quantitative imaging descriptors in line with advances in image processing techniques [12, 13]. High-dimensional imaging data allows an in-depth characterization of tumor phenotypes, with the underlying hypothesis that imaging reflects not only macroscopic but also the cellular and molecular properties of tissues. Radiomic features are complementary to biopsies and have the advantage of being noninvasive, which allows the evaluation of a tumor and its microenvironment, characterization of spatial heterogeneity, and longitudinal assessment of disease evolution [13, 14]. Since radiomic features are composed of hundreds of variables, a predictive modeling algorithm can be implemented for regularization, feature selection, and tree-based algorithms.

Accordingly, we have conducted a study to demonstrate the feasibility of radiomic prediction of the immune microenvironment by using computed tomographic (CT) imaging in NSCLC. Our ultimate goal was to identify useful predictive radiomic characteristics of the immune microenvironment and to further develop treatment strategies.

## Materials and methods

### Datasets

Two independent datasets were used for the training and test sets. The training dataset labeled “Lung3”, contains data from patients treated at the MAASTRO Clinic, the Netherlands [13]. The test set was a dataset from The Cancer Genome Atlas (TCGA), which included both squamous cell carcinoma and adenocarcinoma cases [15, 16]. Both training and test sets were publicly available CT image and gene expression data. Thus, no Institutional Review Board approval specific to this study was required.

### Radiomic feature extraction for prediction variables

**Fig 1:**
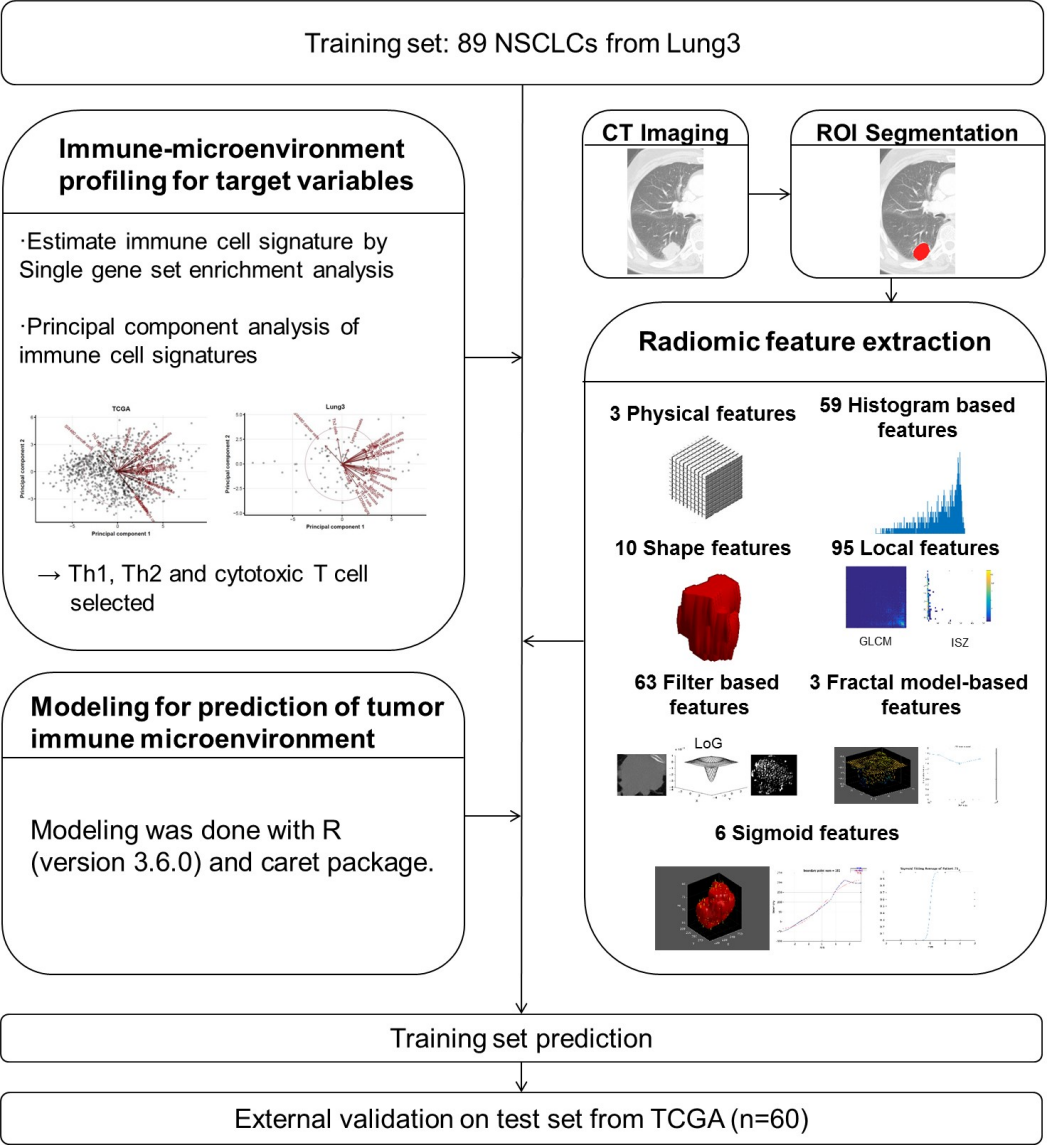
Development of the tumor microenvironment prediction model.

CT images were gathered from The Cancer Imaging Archive (TCIA, http://cancerimagingarchive.net/) [17]. All images from TCIA were acquired using CT scans at following variable image resolutions; For “Lung3”, mean in-plane resolution, 0.913 ± 0.204 mm (range, 0.602–1.367 mm) and mean slice thickness, 3.989 ± 1.236 mm. For “TCGA”, mean in-plane resolution, 0.718 ± 0.136 mm (range, 0.547–0.977 mm) and mean slice thickness, 3.930 ± 1.429 mm. The values following the mean are standard deviation (SD) values. In "Lung 3" cohort, 62 (69.7%) cases were scanned by contrast-enhanced CT and 27 (30.3%) patients were scanned by non-contrast CT. In "TCGA", 25 (41.7%) cases were scanned by contrast-enhanced CT and 35 (58.3%) cases were scanned by non-contrast CT. For the radiomic analysis, regions of interest (ROIs) were delineated on axial CT images to generate a volume of interest that included the entire target lesion (Fig 1). The quantitative CT analysis was based on physical, histogram-based, shape, local, filter-based, and fractal model-based features from the manually derived ROI. All radiomic features were calculated using the open-source code (PyRadiomics) [18] and in-house code using MATLAB. Features unavailable in PyRadiomics were implemented using the in-house code and there are several published articles which have been used the same software [19, 20]. Details are described in S1 File.

### Immune microenvironment profiling for target variables

**Fig 2:**
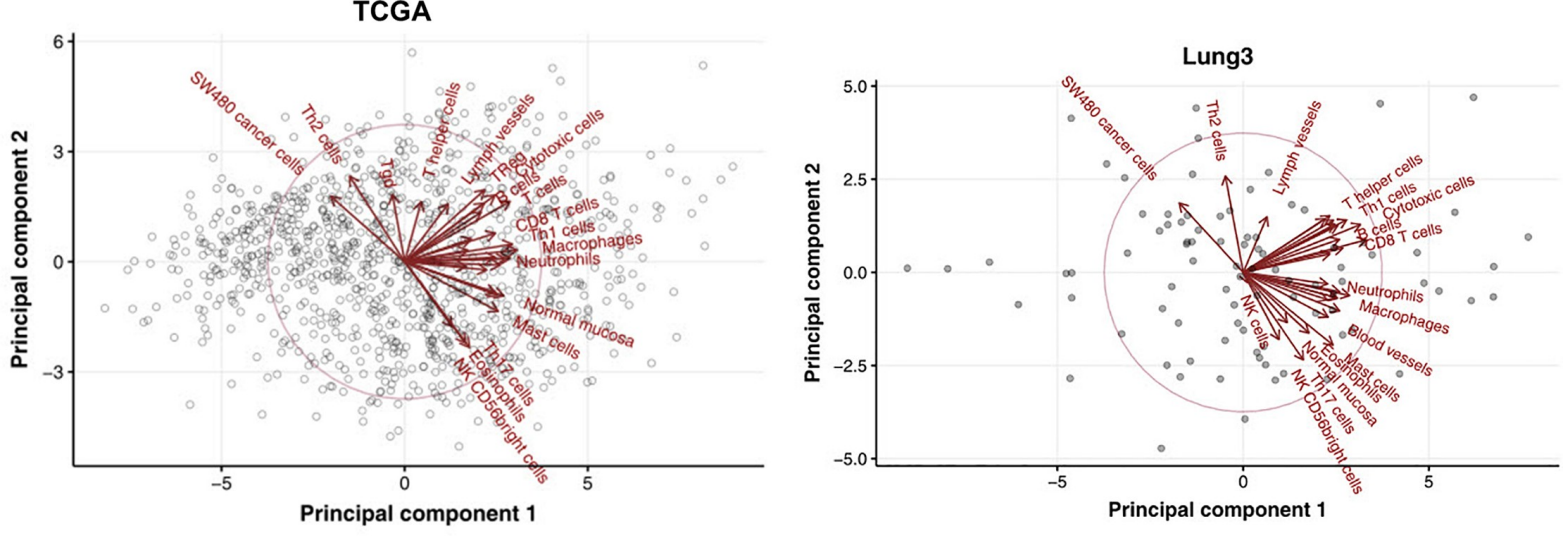
Principal component analysis of immune cell signatures with 1016 TCGA (A) and “Lung3” (B) non-small cell carcinomas.

**Fig 3:**
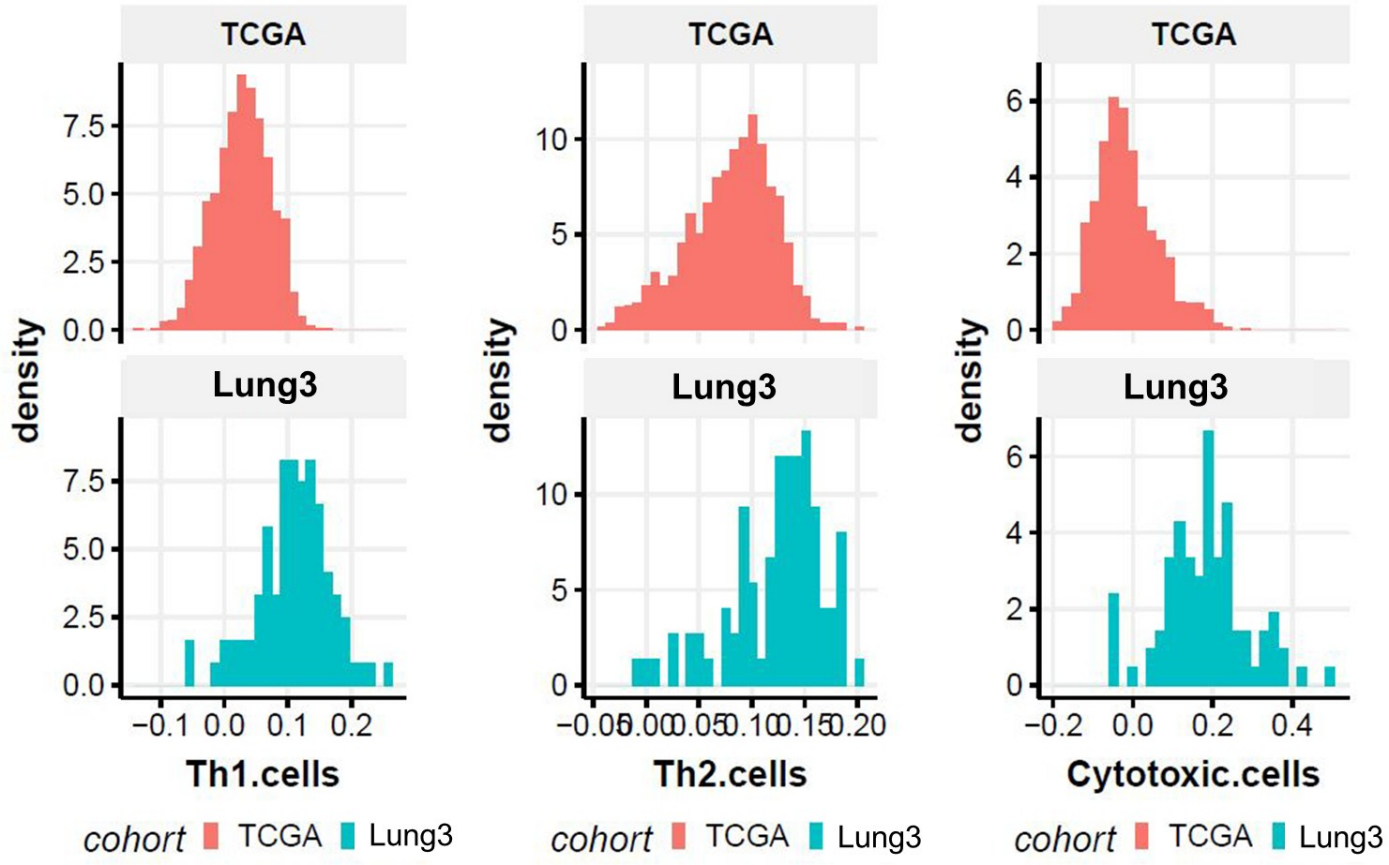
Distributions of type 1 helper T-cell, type 2 helper T-cell, and cytotoxic T cell signatures of the test set (TCGA cohort) and training set (“Lung3” cohort).

The training set gene expression data were generated using the Rosetta/Merck Human RSTA Custom Affymetrix 2.0 microarray (GPL15048) and stored in the Gene Expression Omnibus (GEO accession: GSE58661). The test set gene expression data were generated using RNAseq and downloaded from the Broad GDAC Firehose. The immune cell signature was estimated from gene expression data using single-sample gene set enrichment analysis (ssGSEA) to determine the immune microenvironment of lung cancer. The GSVA R package was used for the ssGSEA [21]. A set of 28 immune cell gene lists was selected from a previous study conducted by Bindea et al. [22]. We focused on Th1, Th2, and cytotoxic T cell signatures. The immune cell signature of TCGA data was initially obtained from all 1016 cases. The test set included 60 TCGA cases that were available with CT images. Principal component analysis (PCA) of immune cell signatures was done with 1016 TCGA non-small cell carcinomas (Fig 2A). The immune cell signature that gave a positive principal component 1 (PC1) score included Th1 cells, CD8 T cells, macrophages, and dendritic cells. The immune cell signature that gave PC2 positive and PC1 negative scores included Th2 cells and cancer cells. The immune cell signature that gave PC2 negative and PC1 positive scores included Th17 cells, eosinophils, and NK CD56 bright cells. This result means that the Th1 cell and Th2 cell signatures are almost independent. The PCA results with “Lung3” were similar to TCGA (Fig 2B). Thus, signatures of Th1, Th2, and cytotoxic T cells were selected as target variables and grouped by their mean cut-off values (high vs. low). The cut-off value was set as the average of the value of immune cell signatures. The training set and the test set applied their own average cut-off value instead of the same cut-off value, because the distributions of Th1, Th2, and cytotoxic T cells signature of the test sets (TCGA cohort) shift further to the right than the training set (“Lung3” cohort) (Fig 3).

### Modeling for the prediction of tumor immune microenvironment

The Th1, Th2, and cytotoxic T cell groups were predicted using machine learning algorithms, which included penalized logistic regression, penalized discriminant analysis, sparse discriminant analysis, linear discriminant analysis, naive Bayes, classification and regression tree (CART), bagged CART, and random forest. R (version 3.6.0) and the caret R package were used for the prediction modeling [23]. Data pre-processing methods included removing zero variance variables, NA imputation (KNN), resolving skewness (Yeo-Johnson), centering, and scaling (Z-score normalization). Data preprocessing was applied to all predictor variables with the recipe (v.0.1.9) rpackage. The detailed preprocessing parameters set with the default value. Resampling was done using the 0.632 bootstrap estimator.

## Results

Demographic information and tumor characteristics for both the training and test sets are presented in Table 1. The training dataset comprised 89 NSCLCs and the test set included 60 cases from all 1016 lung squamous cell carcinoma and adenocarcinomas whose CT images are available. For radiomic feature extraction, we evaluated a total of 239 CT radiomic features, which were divided into seven groups as follows: three physical features, 59 histogram-based features, 10 shape features, 95 local features, 63 filter-based features (LoG filter), three fractal model-based features, and six sigmoid features. Details are provided in S1 Table.

**Table 1:** Demographic information of the “Lung3” and TCGA dataset.

	Lung3 (n = 89)	n	TCGA (n = 60)	n
Age ± SD	N/A		67.3 ± 10.3	60
Sex		89		60
Male	60 (67.4)		28 (46.7)	
Female	29 (32.6)		32 (53.3)	
Stage		87		57
I	35 (40.2)		23 (40.4)	
II	34 (39.1)		18 (31.6)	
III	13 (14.9)		14 (24.6)	
IV	5 (5.7)		2 (3.5)	
Histological subtype		89		60
Squamous cell carcinoma	36 (40.5)		35 (58.3)	
Adenocarcinoma	44 (49.4)		25 (41.7)	
Other	9 (10.1)		0 (0)	
Race	N/A			59
Black or African-American			7 (11.9)	
White			52 (88.1)	
Ethnicity	N/A			57
Not Hispanic or Latino			57 (100)	

TCGA, The Cancer Genome Atlas; SD, standard deviation; N/A, not available

data in parentheses are percentages.

### Training set prediction

**Fig 4:**
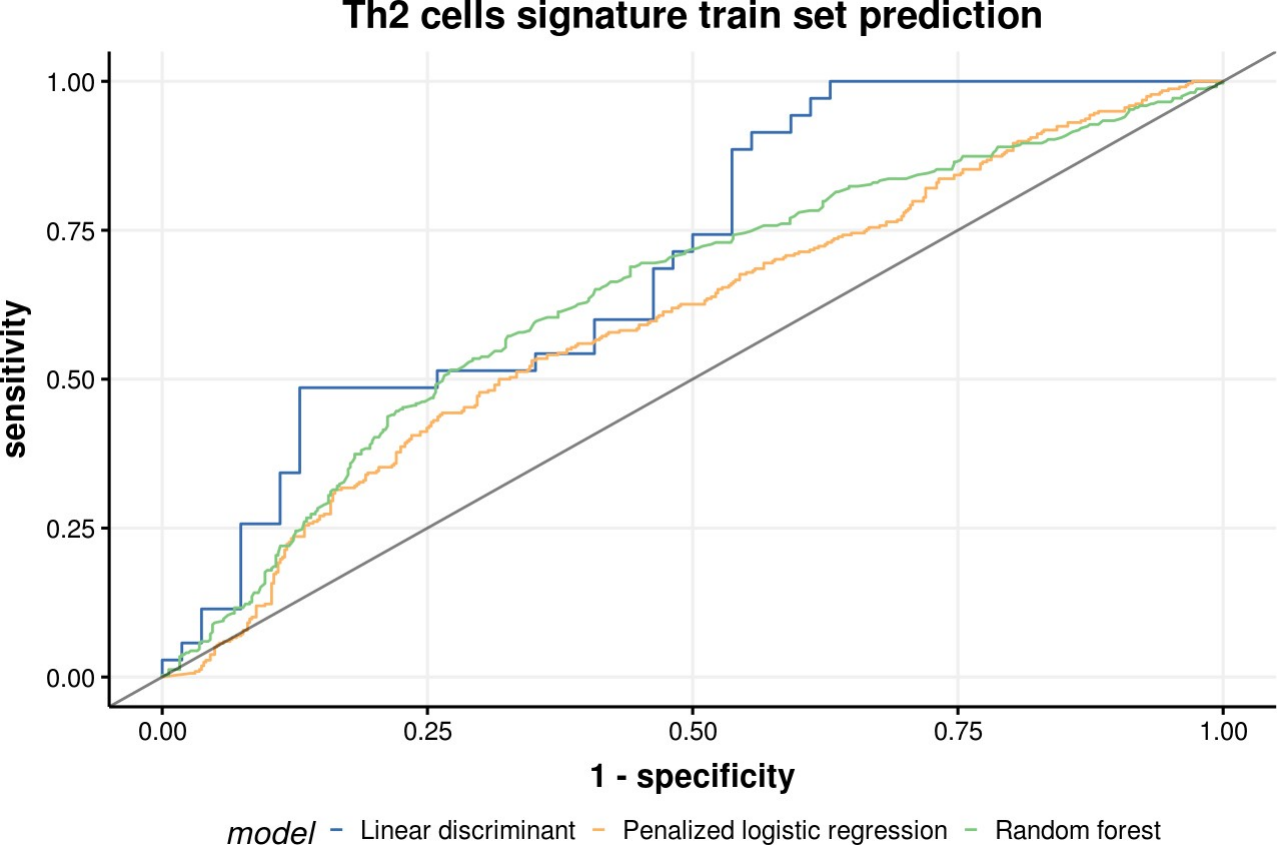
Receiver operating characteristic curve of top 3 models on the training set of type 2 helper T cells.

Classification trees and rule-based models and recursive feature elimination methods performed better than penalized linear classification models. Random forest, linear discriminant analysis, and penalized logistic regression models performed well in the training set of Th2 cell prediction (area under the curve, AUC = 0.795, 0.772, and 0.754, respectively) (Table 2 and Fig 4). Random forest, penalized discriminant analysis, and bagged CART models performed well in the training set of Th1 cells (AUC = 0.751, 0.711, and 0.741, respectively) and cytotoxic T cells (AUC = 0.681, 0.674, and 0.647, respectively) predictions (Tables 3 and 4).

**Table 2:** Type 2 helper T-cell signature training set prediction.

AUC	Sens	Spec	AUCSD	SensSD	SpecSD	Model
0.795	0.642	0.869	0.108	0.194	0.114	Random forest
0.772	0.500	0.840	0.090	0.208	0.192	Linear discriminant
0.754	0.636	0.808	0.08	0.152	0.095	Penalized logistic regression
0.753	0.624	0.832	0.097	0.173	0.136	Bagged CART
0.736	0.546	0.796	0.093	0.119	0.098	Sparse discriminant analysis
0.729	0.633	0.730	0.116	0.276	0.230	Naive Bayes
0.717	0.613	0.740	0.091	0.199	0.138	CART
0.701	0.666	0.762	0.114	0.209	0.126	Penalized discriminant analysis

AUC, area under the curve; Sens, sensitivity; Spec, specificity; AUCSD, area under the curve standard deviation; SensSD, sensitivity standard deviation; SpecSD, specificity standard deviation; CART, classification and regression tree

**Table 3:** Type 1 helper T-cell signature training set prediction.

AUC	Sens	Spec	AUCSD	SensSD	SpecSD	Model
0.751	0.666	0.788	0.083	0.145	0.133	Random forest
0.741	0.679	0.772	0.092	0.155	0.098	Bagged CART
0.711	0.677	0.712	0.087	0.115	0.111	Penalized discriminant analysis
0.709	0.540	0.755	0.165	0.328	0.213	Naive Bayes
0.686	0.598	0.669	0.082	0.111	0.117	Sparse discriminant analysis
0.682	0.646	0.741	0.090	0.111	0.115	Penalized logistic regression
0.676	0.589	0.740	0.084	0.168	0.134	CART
0.606	0.490	0.665	0.204	0.270	0.181	Linear discriminant analysis

AUC, area under the curve; Sens, sensitivity; Spec, specificity; AUCSD, area under the curve standard deviation; SensSD, sensitivity standard deviation; SpecSD, specificity standard deviation; CART, classification and regression tree

**Table 4:** Cytotoxic T cell signature training set prediction.

AUC	Sens	Spec	AUCSD	SensSD	SpecSD	Model
0.681	0.603	0.737	0.064	0.155	0.149	Random forest
0.674	0.604	0.665	0.076	0.144	0.088	Penalized discriminant analysis
0.647	0.597	0.717	0.052	0.130	0.144	Bagged CART
0.628	0.616	0.677	0.074	0.117	0.113	Penalized logistic regression
0.622	0.472	0.696	0.099	0.123	0.137	CART
0.607	0.579	0.586	0.075	0.121	0.121	Sparse discriminant analysis
0.574	0.515	0.625	0.249	0.302	0.246	Linear discriminant
0.545	0.420	0.545	0.167	0.242	0.222	Naive Bayes

AUC, area under the curve; Sens, sensitivity; Spec, specificity; AUCSD, area under the curve standard deviation; SensSD, sensitivity standard deviation; SpecSD, specificity standard deviation; CART, classification and regression tree

### Test set prediction

**Fig 5:**
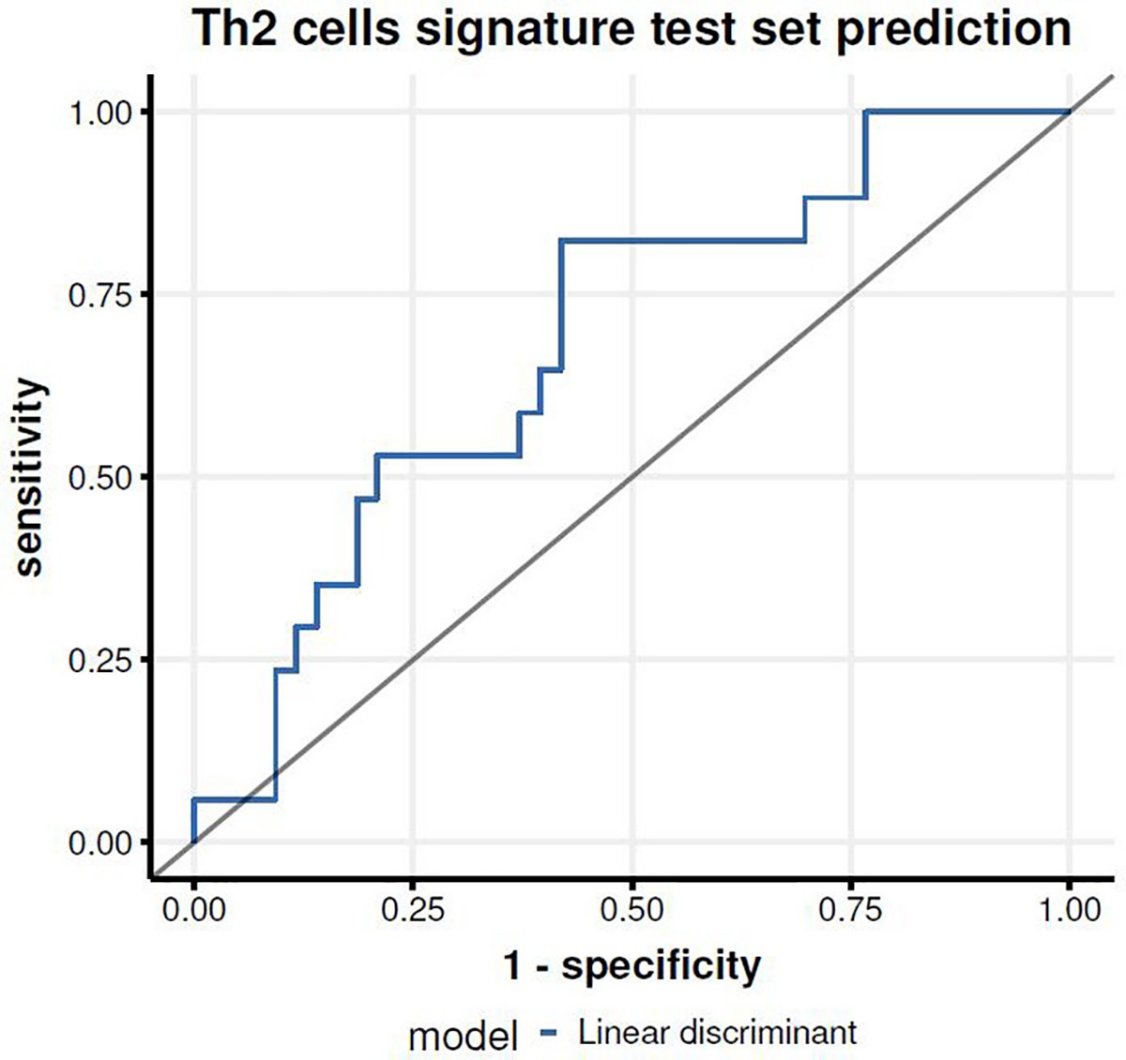
Receiver operating characteristic curve of top 3 models on the test set of type 2 helper T cells.

We selected a linear discriminant analysis model for Th2 cell prediction and penalized discriminant analysis model for Th1 cell prediction because both models are simpler than bagged CART or random forest. Although the tree based ensemble methods including random forest and bagged CART showed best performance, but the performance was not significantly different between the ensemble methods and linear models including linear discriminant analysis and penalized discriminant analysis models. The prediction performance on the test set of Th2 cells was good (AUC = 0.684) (Table 5 and Fig 5) but not on Th1 cells (AUC = 0.536–0.564) and cytotoxic T cells (AUC = 0.533–0.612) (S2 and S3 Tables). Predictors of linear discriminant analysis model were skewness (total and outer pixels), kurtosis, variance (subsampled from delta [subtraction inner pixels from outer pixels]), and informational measure of correlation (IMC) (Table 6).

**Table 5:** Performance of prediction on test set of type 2 helper T-cells.

Model	AUC	*p*-value
Random forest	0.707	0.013
Linear discriminant analysis	0.684	0.027
Sparse discriminant analysis	0.687	0.034

AUC, area under the curve

**Table 6:** Linear discriminant model predictor variables for type 2 helper T-cells.

	Skewness	Skewness (out)	Kurtosis	Variance (deltaS)	IMC
Low	0.543	0.527	-0.498	-0.49	-0.426
High	-0.352	-0.341	0.323	0.317	0.276

IMC, informational measure of correlation

out = outer pixels, delta = inner pixels subtracted from outer pixels, S = subsampled

## Discussion

Several previous reports suggest that malignant phenotypes and efficacy of immunotherapy in cancers are defined not only by the intrinsic activities of cancer cells but also by components in the tumor microenvironment, especially tumor-infiltrating immune cells [24–30]. Despite the volume of previous research in this setting, the complex interactions between tumors and their microenvironment remain poorly understood. In addition, these studies have evaluated tumor-infiltrating immune cells using immunohistochemistry-based analyses alone. Most histologic approaches only involve small biopsies or surgical specimens and are therefore limited due to the heterogeneity of tumors. Thus, there is no valid noninvasive method that describes the whole tumor microenvironment, raising a compelling need for the development of a “translator” to predict the tumor microenvironment. In this situation, radiomics could offer an alternative. However, there are very few studies that have related radiomic analysis to the tumor microenvironment [31, 32].

In this study, based on the deconvolution of gene expression data from 89 patients in the “Lung3” cohort using ssGSEA and immune microenvironment profiling with the use of 28 immune cell signatures from gene expression, we found associations with certain radiomic features for subsets of Th1, Th2, and cytotoxic T cells in NSCLCs. Through a prediction performance test using a TCGA test set, we also demonstrated that the radiomic prediction for Th2 cell signatures of NSCLCs was feasible (AUC = 0.684), even though the performances of radiomics on the test set of Th1 and cytotoxic T cells were not accurate enough to be predictable. Our results indicate that skewness (total and outer pixels), kurtosis, variance (subsampled from delta), and IMC correlated with Th2 cells. Our study is in line with but distinguishable from previous reports [31–33]. The other reports showed an association between radiomics and certain immune cell signatures such as CD8 cell, CD3 cell, or tumor PD-L1 expression, and demonstrated model creation and validation in independent cohorts. And Grossmann at al. [33] used the same data set (“Lung3”) for validation cohort, and found a relationship between radiomic features, immune response, inflammation, and survival, which was further validated by immunohistochemical staining. As in our study, they showed that imaging can provide a promising way to predict the immune phenotype of tumors and to infer clinical outcomes for patients with cancer who had been treated with immunotherapy. Compared with these studies, our study had distinguishable points in that we used more integral radiomic features including filter-based and fractal model-based features reflecting the characteristics of tumor margins, as well as first and second-order or textural features, and estimated the comprehensive immune microenvironment with 28 immune cell signatures. In addition, we homogenized our data by using only NSCLC cases whereas previous two studies dealt with heterogeneous cohorts of different tumor histopathology and location [31, 32]. Even though our results do not provide good generalization across tumor types and locations, they predict the immune microenvironment from radiomic information concentrated on NSCLC, in particular, and therefore we believe our results are more specific.

Most past studies dealing with antitumor immunity have focused on CD8+ cytotoxic T cells, as their cytotoxic activity was deemed to be critical for tumor eradication. Recent work indicates that specific subsets of CD4+ T cells (namely, Th1 cells), B cells, macrophages as well as dendritic cells provide an important contribution to antitumor immune responses [28, 29]. These cytotoxic T cells and Th1 cells are the main cell signatures of the type 1 immune response [9]. In addition, other studies have demonstrated that the levels of Th2 and regulatory T (Treg) cells, which are important elements of the type 2 immune response, are associated with negative clinical outcomes [30]. In contrary to this traditional scheme of pro- and antitumor microenvironment, a recent study demonstrated that the type 2 immune response characterized by Th2 cells may inhibit tumor formation in melanoma [10]. In line with this research, the Th1, Th2, and the cytotoxic T cell signature prediction test and the demonstration that the radiomic prediction of the Th2 cell signature is feasible in our study are clinically meaningful because these three cells are subsets of influential immune cells in tumor immune responses as mentioned above.

We found that skewness (total and outer pixels), kurtosis, variance (subsampled from delta), and IMC were correlated with Th2 cells. Among these features, outer pixel skewness refers to skewness of the peripheral portion of the tumor. More interestingly, given that this radiomic feature reflects the peritumoral radiologic phenotype, this finding may indicate that the immune microenvironment of NSCLCs is closely related with certain interactions between the tumors and surrounding normal lung tissues. In other words, these edges or peritumoral radiologic findings offer information that can help predict tumor immune activity in the development of immunotherapy strategies. There has been no previous research regarding the relationship between the five radiomic features, including skewness of outer pixels and subsets of immune cells, therefore further verification of this finding in larger cohorts is needed.

Despite the advantages of utilizing a different cohort for external validation, this analysis has several limitations. First, the training data are retrospective and limited to 89 patients from a single institution. This deficiency could be addressed in future work by using a larger patient cohort. Second, CT scans we obtained for radiomic feature selection were partly contrast-enhanced and partly non-contrast and there are differences in image resolution and even may be in acquisition protocol between training and test data (Scan protocol including tube voltage and tube current was not provided). Contrast agent may obscure the radiomic textural features and variable acquisition protocol might lead to be unstable in features because textural- and intensity-based features may be affected by intensity and scanner variability. However, it is rather difficult to have the same acquisition protocol between multi-center data in practice. Testing using independent data with some differences in acquisition reflects the real clinical practice and the performance measured under such circumstance is a good measure of generalization of the proposed method. The data are obtained from the public database and many studies using the data still used the data as a whole despite the heterogeneity [13, 34, 35]. Nevertheless, further verification for reproducibility of radiomic features according to acquisition protocol is needed. Lastly, this study includes multiple histologic types in both the “Lung3” and TCGA cohorts. The histologic type could serve as a confounding factor when predicting tumor immune microenvironment status using radiomic features. However, we believe our findings and the comprehensive radiomics approach described herein are meaningful in terms of building baseline research data for the next relevant study. In addition, this study shows the usefulness of radiomics in the tumor microenvironment domain, especially for NSCLCs. Above all, we attempted to perform external validation and our results were favorable for Th2 cell signature prediction, which is clinically important.

## Conclusions

In conclusion, radiomic prediction for the immune microenvironment of NSCLC was feasible and found a relationship between radiomic features and Th2 cell signatures. This study demonstrated the potential of radiomic features as noninvasive biomarkers to capture the tumor microenvironment properties and to predict the tumor-suppressive or tumor-permissive status of NSCLC. The results of this study may help define categories of tumor immune activity for patients with NSCLC and develop immunotherapy strategies.

## Supporting information

S1 File Details about the different groups of radiomic features assessed in our study. (DOCX)

S1 Table Radiomic features used to predict the tumor microenvironment. (DOCX)

S2 Table Performance of prediction on the test set of type 1 helper T cells. (DOCX)

S3 Table Performance of prediction on the test set of cytotoxic T cells. (DOCX)
