# Supplementary material for: Deciphering the tumor microenvironment through radiomics in non-small cell lung cancer: Correlation with immune profiles
Source: PLoS One. 2020 Apr 6;15(4):e0231227. doi: 10.1371/journal.pone.0231227 (PMC7135211; doi:10.1371/journal.pone.0231227)
Supplement: S1 File — (DOCX) [file pone.0231227.s001.docx]

**S1 File.**

In this study, we explored a feature-based approach to extraction and quantification of meaningful and reliable information from computed tomographic (CT) images. In this section, we describe in detail the different groups of radiomic features assessed in our study. We evaluated a total of 239 CT radiomic features, which were divided into seven groups as follows:

1. Physical features
2. Histogram-based features
3. Shape features
4. Local features
5. Filter-based features (LoG filter)
6. Fractal model-based features
7. Sigmoid features

***Group 1. Physical features***

Let $R$ denote the 3D image resolution,$V$denote the tumor volume, and $D$ denote the tumor density.

- 1. **Volume:**
  $volume=R*number of voxels$
  2. **Mass:**
     $mass=V*D$
  3. **Density:**

Physical density (g/cm^3^) was estimated by extrapolating from the mean CT

scan attenuation.

***Group 2. Histogram-based features***

Histogram-based features describe the distribution of voxel intensities within the region of interest (ROI) of the CT image through commonly used basic metrics. Let $X$ denote the three-dimensional image matrix with $N$ voxels and $P$ the first-order histogram with $N_{l}$ discrete intensity levels.

- 1. **Energy:**
  $energy=\sum_{i}^{N} {X(i)}^{2}$
  2. **Entropy:** $entropy=-\sum_{i=1}^{N_{l}} P(i)\log_{2} P(i)$
  3. **Interquartile range:**

The interquartile range of intensity values of .

- 1. **Kurtosis:** $kurtosis=\frac{\frac{1}{N}\sum_{i=1}^{N} {(X\left( i \right)-\bar{X})}^{4}}{\left( \sqrt{\frac{1}{N}\sum_{i=1}^{N} \left( X\left( i \right)-\bar{X} \right)^{2}} \right)^{2}}$ where $\bar{X}$ is the mean of $X$.
  2. **Maximum:**The maximum intensity value of $X$.
  3. **Mean:** $mean=\frac{1}{N}\sum_{i}^{N} X(i)$
  4. **Median:**The median intensity value of $X$.
  5. **Minimum:**The minimum intensity value of $X$.
  6. **Range:**The range of intensity values of $X$.
  7. **Root mean square (RMS):**
     $RMS=\sqrt{\frac{\sum_{i}^{N} {X(i)}^{2}}{N}}$
  8. **Skewness:** $skewness=\frac{\frac{1}{N}\sum_{i=1}^{N} {(X\left( i \right)-\bar{X})}^{3}}{\left( \sqrt{\frac{1}{N}\sum_{i=1}^{N} {(X\left( i \right)-\bar{X})}^{2}} \right)^{3}}$ where $\bar{X}$ is the mean of $X$.
  9. **Standard deviation:**

$$standard deviation=\left( \frac{1}{N-1}\sum_{i=1}^{N} {(X\left( i \right)-\bar{X})}^{2} \right)^{1/2}$$

where $\bar{X}$ is the mean of .

- 1. **Uniformity:**
$uniformity=\sum_{i=1}^{N_{l}} {P(i)}^{2}$
  2. **Variance:** $variance=\frac{1}{N-1}\sum_{i=1}^{N} {(X\left( i \right)-\bar{X})}^{2}$ where $\bar{X}$ is the mean of $X$.

The standard deviation and variance are measures of histogram dispersion, that is, a measure of how much the gray levels differ from the mean. The variance, skewness, and kurtosis are the most frequently used central moments. The skewness measures the degree of histogram asymmetry around the mean, and kurtosis is a measure of histogram sharpness. As measures of histogram randomness, we computed the uniformity and entropy of the image histogram. From the histogram, CT numbers or Hounsfield units (HUs) at the 2.5th, 25th, 50th, 75th, and 97.5th percentiles were also computed.

***Group 3. Shape features***

In this group of features, we included descriptors of the three-dimensional size and shape of the tumor region. Let $V$ denote the volume and $A$ the surface area of the volume of interest. We computed the following shape- and size-based features:

- 1. **Compactness**:
  $compactness=\frac{V}{\sqrt{\pi}A^{\frac{2}{3}}}$
  2. **Convexity:**

Measures ratio of the ROI volume contained within the tumor to the calculated convex hull volume.

$$Convexity=\frac{V}{V'}$$

where $V$ denotes tumor volume and $V^{'}$ denotes convex hull volume [1].

- 1. **Eccentricity:**

A measure of how close a shape is to an ellipse on the 2D image. 

$$Eccentricity=c/a$$

where $c$ is the distance from the center to a focus and $a$ is the distance from that focus to a vertex [2].

- 1. **Maximum 3D diameter:**

The maximum three-dimensional tumor diameter is measured as the largest pairwise Euclidean distance between voxels on the surface of the tumor volume.

- 1. **Roundness factor:**

A measure of convexity of a tumor’s profile on the 2D image.

$$RF=\frac{4\pi\cdot Area}{Perimeter^{2}}$$

where $Area$ is the area of the tumor and $Perimeter$ the length of tumor contour on the 2D image.

- 1. **Solidity:**

A measure of convexity of a tumor’s profile on the 2D image.

$$Solidity=\frac{Area}{Convex area}$$

where $Area$ is the area of the tumor and $Convex area$ denotes the area of the convex hull at the boundary of the tumor on the 2D image.

- 1. **Spherical disproportion**:

$$spherical disproportion=\frac{A}{4\pi R^{2}}$$

where $R$ is the radius of a sphere with the same volume as the tumor.

- 1. **Sphericity**:
$sphericity=\frac{\pi^{\frac{1}{3}}{(6V)}^{\frac{2}{3}}}{A}$
  2. **Surface area:**

The surface area is calculated by triangulation (i.e. dividing the surface into connected triangles) and is defined as

$$A=\sum_{i=1}^{N} \frac{1}{2}\left| a_{i}b_{i}\times a_{i}c_{i} \right|$$

where $N$ is the total number of triangles covering the surface and $a$, $b,$ and $c$ are edge vectors of the triangles.

- 1. **Surface to volume ratio**:
$surface to volume ratio=\frac{A}{V}$

The maximum 3D diameter and surface area provide information on the size of the lesion. Measures of compactness, convexity, eccentricity, roundness factor, spherical disproportion, sphericity, and surface to volume ratio describe how spherical, rounded, or elongated the shape of the tumor is.

***Group 4. Local features***

The features from group 2 (histogram-based features) provide information related to the gray-level distribution of the image. However, they do not provide any information regarding the relative positions of the various gray levels over the image. In this group, we therefore included local features describing patterns or the spatial distribution of voxel intensities, which were calculated from gray-level co-occurrence (GLCM)[3], gray level size zone (GLSZM)[4], and neighborhood gray tone difference (NGTDM)[5] texture matrices. Texture matrices were determined using 26-connected voxels (i.e., voxels considered to be neighbors in all 13 directions in three dimensions).

*Gray-level co-occurrence matrix-based features*

A GLCM is defined as $P(i,j;\delta,\alpha)$, a matrix with size $N_{g}\times N_{g}$ describing the second-order joint probability function of intensity occurrence pairs of an image, where the $(i,j)$th element represents the number of times the combination of intensity levels $i$ and $j$ occur in two pixels in the image, that are separated by a distance of $\delta$ pixels in direction $\alpha$, and $N_{g}$ is the number of discrete gray-level intensities. As a two-dimensional example, let the following matrix represent a 5 × 5 image, having five discrete gray levels:

$$I=\begin{matrix} 1 & 2 & 5 & 2 & 3 \\ 3 & 2 & 1 & 3 & 1 \\ 1 & 3 & 5 & 5 & 2 \\ 1 & 1 & 1 & 1 & 2 \\ 1 & 2 & 4 & 3 & 5 \end{matrix}$$

For distance $\delta=1$ (considering pixels with a distance of 1 pixel from each other) in direction $\alpha=0$, where 0 degrees is the horizontal direction, the following GLCM is obtained:

$$P(1,0)=\begin{matrix} 3 & 3 & 2 & 0 & 0 \\ 1 & 0 & 1 & 1 & 1 \\ 0 & 1 & 0 & 0 & 2 \\ 0 & 0 & 1 & 0 & 0 \\ 0 & 2 & 0 & 0 & 1 \end{matrix}$$

In this study, distance $\delta$ was set to 1 and direction $\alpha$ to each of the 13 directions in three dimensions, yielding a total of 13 GLCMs for each 3D image. The average of 13 matrices was used as the final GLCM. We then computed various GLCM features from the final GLCM.

Let:

$P(i,j)$ be the co-occurrence matrix for an arbitrary $\delta$ and $\alpha$,

$N_{g}$ be the number of discrete intensity levels in the image,

$\mu$ be the mean of $P(i,j)$,

$p_{x}\left( i \right)=\sum_{j=1}^{N_{g}} P(i,j)$ be the marginal row probabilities,

$p_{y}\left( i \right)=\sum_{i=1}^{N_{g}} P(i,j)$ be the marginal column probabilities,

$\mu_{x}$ be the mean of $p_{x}$,

$\mu_{y}$ be the mean of $p_{y}$,

$\sigma_{x}$ be the standard deviation of $p_{x}$,

$\sigma_{y}$ be the standard deviation of $p_{y}$,

$p_{x+y}\left( k \right)=\sum_{i=1}^{N_{g}} \sum_{j=1}^{N_{g}} P\left( i,j \right)$, $i+j=k$, $k=2,3,\ldots,2N_{g}$,

$p_{x-y}\left( k \right)=\sum_{i=1}^{N_{g}} \sum_{j=1}^{N_{g}} P\left( i,j \right)$, $\left| i-j \right|=k$, $k=0,1,\ldots,N_{g}-1$,

$HX=-\sum_{i=1}^{N_{g}} p_{x}(i)\log_{2} \left[ p_{x}(i) \right]$ be the entropy of $p_{x}$,

$HY=-\sum_{i=1}^{N_{g}} p_{y}(i)\log_{2} \left[ p_{y}(i) \right]$ be the entropy of $p_{y}$,

$H=-\sum_{i=1}^{N_{g}} \sum_{j=1}^{N_{g}} P\left( i,j \right)\log_{2} \left[ P\left( i,j \right) \right]$ be the entropy of ,

,

.

- 1. **Autocorrelation:**
  $autocorrelation=\sum_{i=1}^{N_{g}} \sum_{j=1}^{N_{g}} ijP(i,j)$
  2. **Cluster Tendency:**
     $cluster tendency=\sum_{i=1}^{N_{g}} \sum_{j=1}^{N_{g}} \left[ i+j-\mu_{x}\left( i \right)-\mu_{y}\left( j \right) \right]^{2}P(i,j)$
  3. **Contrast:**
     $contrast=\sum_{i=1}^{N_{g}} \sum_{j=1}^{N_{g}} \left| i-j \right|^{2}P(i,j)$
  4. **Difference entropy:**
     $difference entropy=\sum_{i=0}^{N_{g}-1} P_{x-y}(i)\log_{2} \left[ P_{x-y}(i) \right]$
  5. **Dissimilarity:**
     $dissimilarity=\sum_{i=1}^{N_{g}} \sum_{j=1}^{N_{g}} \left| i-j \right|P(i,j)$
  6. **Energy:**
     $energy=\sum_{i=1}^{N_{g}} \sum_{j=1}^{N_{g}} \left[ P(i,j) \right]^{2}$
  7. **Entropy:**
     $entropy=-\sum_{i=1}^{N_{g}} \sum_{j=1}^{N_{g}} P\left( i,j \right)\log_{2} \left[ P\left( i,j \right) \right]$
  8. **Homogeneity:**
     $homogeneity=\sum_{i=1}^{N_{g}} \sum_{j=1}^{N_{g}} \frac{P(i,j)}{1+\left| i-j \right|}$
  9. **Informational measure of correlation (IMC):**
     $IMC=\frac{HXY-HXY1}{\text{max}\left\{ HX,HY \right\}}$
  10. **Maximum Probability:**
      $maximum probability=\text{max}\left\{ P(i,j) \right\}$
  11. **Variance:**

$$variance=\sum_{i=1}^{N_{g}} \sum_{j=1}^{N_{g}} {(i-\mu)}^{2}P(i,j)$$

*Intensity size zone matrix-based features*

Intensity size zone metrics (ISZM) quantify size zone matrices in an image. Instead of looking in several directions as in a GLCM, these metrics look at a flat zone size for the whole image. A flat zone is a group of connecting pixels with the same gray level. In ISZM $p(i,j)$, the $(i,j)$th element describes the frequency of matrices of size $j$ with gray level $i$, and $N_{g}$ is the number of discrete gray level intensities. As a 2D example, consider the following 5×5 image with five discrete gray levels:

$$I=\begin{matrix} 5 & 2 & 5 & 4 & 4 \\ 3 & 3 & 3 & 1 & 3 \\ 2 & 1 & 1 & 1 & 3 \\ 4 & 2 & 2 & 2 & 3 \\ 3 & 5 & 3 & 3 & 2 \end{matrix}$$

The ISZM matrix becomes

$$p=\begin{matrix} 0 & 0 & 0 & 1 & 0 \\ 2 & 0 & 1 & 0 & 0 \\ 1 & 1 & 2 & 0 & 0 \\ 1 & 1 & 0 & 0 & 0 \\ 3 & 0 & 0 & 0 & 0 \end{matrix}$$

Let

$p(i,j)$ be the $\left( i,j \right)$th entry in the given size matrix $p,$

$N_{g}$ be the number of discrete intensity values in the image,

$N_{r}$ be the number of different size matrices, and

$N_{p}$ be the number of voxels in the image.

- 1. **Intensity variability (IV)**
  $IV=\frac{\sum_{i=1}^{N_{g}} \left[ \sum_{j=1}^{N_{r}} p(i,j) \right]^{2}}{\sum_{i=1}^{N_{g}} \sum_{j=1}^{N_{r}} p(i,j)}$
  2. **Size zone variability (SZV)**

$$SZV=\frac{\sum_{j=1}^{N_{r}} \left[ \sum_{i=1}^{N_{g}} p(i,j) \right]^{2}}{\sum_{i=1}^{N_{g}} \sum_{j=1}^{N_{r}} p(i,j)}$$

*Neighborhood gray tone difference matrix-based features*

Neighborhood gray tone difference matrix (NGTDM) is a column matrix [5]. It is calculated using neighborhood intensity differences. The $i^{th}$entry of the NGTDM can be denoted$P(i)$, defined as

$$P(i)=\left\{\begin{matrix} \sum_{i\in\left\{\left.N_{i}\right\}\right.} \left|i-\overline{A_{i}}\right|\text{ }if\text{ }N_{i}\text{>0,} \\ 0\text{ }otherwise. \end{matrix}\right.$$

where $\{N_{i}\}$ is the set of all voxels with gray-level $i$ in the tumor volume (including the peripheral region), $N_{i}$ is the number of voxels with gray-level $i$ in the tumor volume, and $A_{i}$ is the average gray level of the $M$ connected neighbors around a center voxel $V(i,j,k)$ with gray level $i$. In this study, we calculated five texture features from NGTDM. The features were defined previously [6] as follows.

- 1. **Coarseness**

High coarseness means that gray-level differences in the

neighborhood are small.

$$coarseness=[\varepsilon+\sum_{i=1}^{N_{g}} n_{i}P(i)]^{-1}$$

where $\varepsilon$ is a small number to prevent coarseness from becoming infinite, and *N_g_* is the number of discrete intensity values in the image.

- 1. **Contrast**

Contrast measures homogeneity in the ROI. A small contrast value means that

the intensity difference between neighborhood pixels is small.

- 1. **Busyness**

Busyness measures how fast the intensity changes in the ROI.

- 1. **Complexity**

Complexity measures the visual information content of a texture in the ROI.

- 1. **Strength**

In general, a high strength value (strong texture) means that texture features are easily defined and clearly visible.

$$strength=\frac{\sum_{i=1}^{N_{g}} \sum_{j=1}^{N_{g}} (n_{i}\text{+}n_{j})(i-j)^{2}}{\varepsilon+\sum_{i=1}^{N_{g}} P(i)},n_{i}\neq0,n_{j}\neq0$$

where $\varepsilon$ is a small number to prevent strength becoming infinite.

***Group 5. Filter-based features (LoG features): Histogram-based features of the Laplacian of Gaussian***

The Laplacian of an image brings out areas of rapid intensity change and is usually used for edge detection. A Gaussian filter is applied prior to the Laplacian to smooth the image and reduce noise.

The equation of LoG with 2D kernels is:

$$LoG\left( x,y \right)=-\frac{1}{{\pi\sigma}^{4}}\left[ 1-\frac{x^{2}+y^{2}}{2\sigma^{2}} \right]e^{-\frac{x^{2}+y^{2}}{2\sigma^{2}}}$$

$$\sigma\epsilon\left[ 0.5 mm:0.5:3.5 mm \right]$$

Texture size (fine to coarse) was specified by modifying the Gaussian radius parameter (from 0.5 to 3.5 mm, in 0.5-mm increments). Histogram-based features (mean, maximum, median, minimum, entropy, uniformity, standard deviation, skewness, and kurtosis) described in Group 2 were investigated.

***Group 6. Fractal model-based features***

**6.1. Fractal dimension (Box-counting):**

The fractal dimension describes the relationship between changes in a measuring scale and measurement results at the scale. In this study, a 3D box-counting algorithm was applied to calculate the fractal dimension to quantify the intensity of tumor homogeneity [2, 7-9].

**6.2. Lacunarity:**

The lacunarity is a measure of the texture or distribution of gaps within an image [8]. It is a counterpart to the fractal dimension and has to do with the size distribution of holes. If a fractal has large gaps or holes, it has high lacunarity; in contrast, if a fractal is almost translationally invariant, it has low lacunarity.

**6.3. Fractal signature dissimilarity**

Fractal signature dissimilarity [10] is defined as the slope of the best linear fit of blanket surface area changes. This feature describes tumor heterogeneity. The blanket method [11] was adopted to calculate the fractal signature dissimilarity.

***Group 7. Sigmoid features***

To quantify tumor margins, the sigmoid function is used to fit density change along a sampling line drawn orthogonal to the tumor surface. Each sampling line, going through one voxel on the tumor surface, has a certain length (3, 5, and 7 mm in this work) inside and outside the tumor.

**References**

1. Grove O, Berglund AE, Schabath MB, Aerts HJ, Dekker A, Wang H, et al. Quantitative computed tomographic descriptors associate tumor shape complexity and intratumor heterogeneity with prognosis in lung adenocarcinoma. PloS one. 2015;10(3):e0118261. Epub 2015/03/05. doi: 10.1371/journal.pone.0118261. PubMed PMID: 25739030; PubMed Central PMCID: PMCPMC4349806.

2. Aerts HJ, Grossmann P, Tan Y, Oxnard GR, Rizvi N, Schwartz LH, et al. Defining a radiomic response phenotype: a pilot study using targeted therapy in NSCLC. Scientific reports. 2016;6:33860. Epub 2016/09/21. doi: 10.1038/srep33860. PubMed PMID: 27645803; PubMed Central PMCID: PMCPMC5028716.

3. Haralick R, Shanmugam K, Dinstein I. Textural features for image classification. IEEE Trans Syst Man Cybern 1973;3(6):610-21.

4. Thibault G, Angulo J, Meyer F. Advanced Statistical Matrices for Texture Characterization: Application to Cell Classification. IEEE Trans Biomed Eng 2014;61(3):630–7.

5. Amadasun M, King R. Textural features corresponding to textural properties. IEEE Trans Sys Man Cyb 1989;19:1264-74.

6. Niu L, Qian M, Yang W, Meng L, Xiao Y, Wong KK, et al. Surface roughness detection of arteries via texture analysis of ultrasound images for early diagnosis of atherosclerosis. PloS one. 2013;8(10):e76880. Epub 2013/10/23. doi: 10.1371/journal.pone.0076880. PubMed PMID: 24146940; PubMed Central PMCID: PMCPMC3798305.

7. Mandelbrot B. How long is the coast of britain? Statistical self-similarity and fractional dimension. Science (New York, NY). 1967;156(3775):636-8. Epub 1967/05/05. doi: 10.1126/science.156.3775.636. PubMed PMID: 17837158.

8. Lennon FE, Cianci GC, Cipriani NA, Hensing TA, Zhang HJ, Chen CT, et al. Lung cancer-a fractal viewpoint. Nature reviews Clinical oncology. 2015;12(11):664-75. Epub 2015/07/15. doi: 10.1038/nrclinonc.2015.108. PubMed PMID: 26169924; PubMed Central PMCID: PMCPMC4989864.

9. Kido S, Kuriyama K, Higashiyama M, Kasugai T, Kuroda C. Fractal analysis of internal and peripheral textures of small peripheral bronchogenic carcinomas in thin-section computed tomography: comparison of bronchioloalveolar cell carcinomas with nonbronchioloalveolar cell carcinomas. Journal of computer assisted tomography. 2003;27(1):56-61. Epub 2003/01/25. PubMed PMID: 12544244.

10. Wang C, Subashi E, Yin FF, Chang Z. Dynamic fractal signature dissimilarity analysis for therapeutic response assessment using dynamic contrast-enhanced MRI. Medical physics. 2016;43(3):1335-47. Epub 2016/03/05. doi: 10.1118/1.4941739. PubMed PMID: 26936718; PubMed Central PMCID: PMCPMC4760981.

11. Paskas MP, Reljin IS, Reljin BD. Multifractal framework based on blanket method. TheScientificWorldJournal. 2014;2014:894546. Epub 2014/03/01. doi: 10.1155/2014/894546. PubMed PMID: 24578664; PubMed Central PMCID: PMCPMC3919058.
