# Supplementary material for: Deciphering the tumor microenvironment through radiomics in non-small cell lung cancer: Correlation with immune profiles
Source: PLoS One. 2020 Apr 6;15(4):e0231227. doi: 10.1371/journal.pone.0231227 (PMC7135211; doi:10.1371/journal.pone.0231227)
Supplement: S1 Table — (DOCX) [file pone.0231227.s002.docx]

| **Supplementary Table 1. Radiomic features used to predict the tumor microenvironment** | |
| --- | --- |
| Type | Lesion Feature |
| **Physical features** | Volume |
|  | Mass |
|  | Density |
| **Histogram-based features** | Mean (total/pp/in/out/delta) |
|  | Standard deviation (total/pp/in/out/delta) |
|  | Variance (total/pp/in/out/delta) |
|  | Maximum (total/pp/in/out/delta) |
|  | Median (total/pp/in/out/delta) |
|  | Minimum (total/pp/in/out/delta) |
|  | Interquartile range (total/pp) |
|  | Range (total/pp) |
|  | Root mean square (total/pp) |
|  | Skewness (total/pp/in/out/delta) |
|  | Energy (total/pp) |
|  | Entropy (total/pp/in/out/delta) |
|  | Kurtosis (total/pp/in/out/delta) |
|  | Uniformity (total) |
|  | HU at the 2.5^th^/25^th^/50^th^/75^th^/97.5^th^ percentile on the histogram |
| **Shape features** | Roundness factor |
|  | Eccentricity |
|  | Solidity |
|  | Convexity |
|  | Surface area |
|  | Compactness |
|  | Max3D diameter |
|  | Spherical disproportion |
|  | Sphericity |
|  | Surface to volume ratio |
| **Local features** | Auto correlation (total/in/out/delta/S/inS/outS/deltaS) |
|  | Cluster tendency (total/in/out/delta/S/inS/outS/deltaS) |
|  | Contrast (total/in/out/delta/S/inS/outS/deltaS) |
|  | Difference entropy (total/in/out/delta/S/inS/outS/deltaS) |
|  | Dissimilarity (total/in/out/delta/S/inS/outS/deltaS) |
|  | Entropy (total/in/out/delta/S/inS/outS/deltaS) |
|  | Energy (total/in/out/delta/S/inS/outS/deltaS) |
|  | Homogeneity (total/in/out/delta/S/inS/outS/deltaS) |
|  | IMC (total/in/out/delta/S/inS/outS/deltaS) |
|  | Max probability (total/in/out/delta/S/inS/outS/deltaS) |
|  | Variance (total/in/out/delta/S/inS/outS/deltaS) |
|  | Intensity variability |
|  | Size zone variability |
|  | Coarseness |
|  | Contrast |
|  | Busyness |
|  | Complexity |
|  | Strength |
| **Filter-based features** | Mean |
| LoG filter | Maximum |
| (σ = 0.5, 1, 1.5, 2, 2.5, 3, 3.5) | Median |
|  | Minimum |
|  | Entropy |
|  | Uniformity |
|  | Standard deviation |
|  | Skewness |
|  | Kurtosis |
| **Fractal model-based features** | Fractal dimension (Box-counting) |
|  | Lacunarity |
|  | Fractal signature dissimilarity |
| **Sigmoid features** | Mean (3/5/7 mm) |
|  | Standard deviation (3/5/7 mm) |

Abbreviations: HU, Hounsfield unit; IMC, Informational measure of correlation; LoG, Laplacian of Gaussian

Note: total = total pixels, pp = positive pixel, in = inner pixels, out = outer pixels, delta = inner pixels subtracted from outer pixels, S = subsampled, inS = subsampled from inner pixels, outS= subsampled from outer pixels, deltaS = subsampled from delta
