# Supplementary material for: Deciphering the tumor microenvironment through radiomics in non-small cell lung cancer: Correlation with immune profiles
Source: PLoS One. 2020 Apr 6;15(4):e0231227. doi: 10.1371/journal.pone.0231227 (PMC7135211; doi:10.1371/journal.pone.0231227)
Supplement: S2 Table — (DOCX) [file pone.0231227.s003.docx]

**Supplementary Table 2. Performance of prediction on the test set of type 1 helper T cells**

| Model | AUC | *p*-value |
| --- | --- | --- |
| Random forest | 0.537 | 0.637 |
| Bagged CART | 0.564 | 0.401 |
| Penalized discriminant analysis | 0.536 | 0.209 |

Abbreviations: AUC, area under the curve; CART, classification and regression tree
