# Supplementary material for: Deciphering the tumor microenvironment through radiomics in non-small cell lung cancer: Correlation with immune profiles
Source: PLoS One. 2020 Apr 6;15(4):e0231227. doi: 10.1371/journal.pone.0231227 (PMC7135211; doi:10.1371/journal.pone.0231227)
Supplement: S3 Table — (DOCX) [file pone.0231227.s004.docx]

**Supplementary Table 3. Performance of prediction on the test set of cytotoxic T cells**

| Model | AUC | *p*-value |
| --- | --- | --- |
| Random forest | 0.612 | 0.139 |
| Penalized discriminant analysis | 0.533 | 0.873 |
| Bagged CART | 0.591 | 0.220 |

Abbreviations: AUC, area under the curve; CART, classification and regression tree
